# Alkylamides of *Acmella oleracea*

**DOI:** 10.3390/molecules20046970

**Published:** 2015-04-16

**Authors:** Yuan-Bin Cheng, Rosa Huang Liu, Meng-Chi Ho, Tung-Ying Wu, Ching-Yeu Chen, I-Wen Lo, Ming-Feng Hou, Shyng-Shiou Yuan, Yang-Chang Wu, Fang-Rong Chang

**Affiliations:** 1 Graduate Institute of Natural Products, College of Pharmacy, Kaohsiung Medical University, Kaohsiung 807, Taiwan; E-Mails: jmb@kmu.edu.tw (Y.-B.C.); usa19871216@yahoo.com.tw (M.-C.H.); Kuma0401@gmail.com (T.-Y.W.); iwenlo99@gmail.com (I.-W.L.); 2 Center for Infectious Disease and Cancer Research, Kaohsiung Medical University, Kaohsiung 807, Taiwan; 3 School of Nutrition, College of Health Care and Management, Chung Shan Medical University, Taichung 40201, Taiwan; E-Mail: rhl@csmu.edu.tw; 4 Chinese Herbal Medicine Research Center, China Medical University, Taichung 404, Taiwan; 5 Department of Physical Therapy, Tzu-Hui Institute of Technology, Pingtung 926, Taiwan; E-Mail: chingyeu1971@yahoo.com.tw; 6 Translational Research Center and Cancer Center, Kaohsiung Medical University Hospital, Kaohsiung 807, Taiwan; E-Mails: mifeho@kmu.edu.tw (M.-F.H.); yuanssf@ms33.hinet.net (S.-S.Y.); 7 School of Pharmacy, College of Pharmacy, China Medical University, Taichung 404, Taiwan; 8 Chinese Medicine Research and Development Center, China Medical University Hospital, Taichung 404, Taiwan; 9 Center for Molecular Medicine, China Medical University Hospital, Taichung 404, Taiwan; 10 Cancer Center, Kaohsiung Medical University Hospital, Kaohsiung 807, Taiwan; 11 Department of Marine Biotechnology and Resources, National Sun Yat-sen University, Kaohsiung 804, Taiwan; 12 Research Center for Natural Product & Drug Development, Kaohsiung Medical University, Kaohsiung 807, Taiwan

**Keywords:** *Acmella oleracea*, alkylamide, quality control assessment

## Abstract

Phytochemical investigation of the flowers of *Acmella oleracea* had resulted in the isolation of one new alkylamide, (2*E*,5*Z*)-*N*-isobutylundeca-2,5-diene-8,10-diynamide (**1**), together with four known analogues (**2**−**5**). The structures of these compounds were determined by the interpretation of spectroscopic methods, especially NMR technologies (COSY, HSQC, HMBC, and NOESY). In addition, a convenient method for concentrating the alkylamide-rich fraction and analyzing fingerprint profile of *A. oleracea* was established.

## 1. Introduction

Plant belonging to genus *Acmella* (Family Asteraceae) is an annual herb native to the tropical parts of Africa and America with a yellow flower head. There are more than 30 species of this genus all over the world. *Acmella oleracea* (syn. *Spilanthes oleracea*, *S. acmella*), the most common cultured species, is commonly used in Africa and India as a traditional folk medicine to cure toothache, throat complaint, stomatitis, and malaria [1]. In a recent pharmaceutical study, the cold water extract of *A. oleracea* flowers show antinociception activity by inhibiting prostaglandin synthesis, interrupting nociception transmission, and exerting antihistamine activity [2]. As a result of earlier phytochemical studies, alkylamides [3,4,5,6], 3-acetylaleuritolic acid, β-sitostenone, scopoletin, vanillic acid, *trans*-ferulic acid, and *trans*-isoferulic acid [7] were isolated as the major constituents of *A. oleracea*. Among these secondary metabolites, alkylamides are regarded for their diuretic [8], antibacterial [9], and anti-inflammatory [10] activities. Besides the medicinal use, alkylamides are well-known for their anti-wrinkle activity and used in many skin care products. The production of *A. oleracea* faces a critical problem due to the lack of a reliable quality assessment method for determining the concentration of alkylamides. To detect concentrations of these bioactive markers, a rapid HPLC-PDA method for simultaneous detection of alkylamides from *A. oleracea* was established by our research group. Furthermore, this method was applied for the detection of alkylamides from different parts of *A. oleracea.* Herein, the isolation and structure elucidation of a new alkylamide, the method for concentrating the alkylamide-rich fraction, and the contents of alkylamide from *A. oleracea* are reported.

## 2. Results and Discussion

**Scheme 1:**
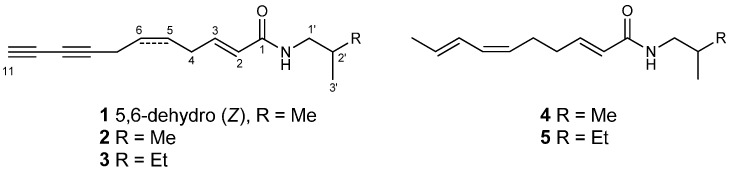
The structures of alkylamides **1−5**.

**Figure 1:**
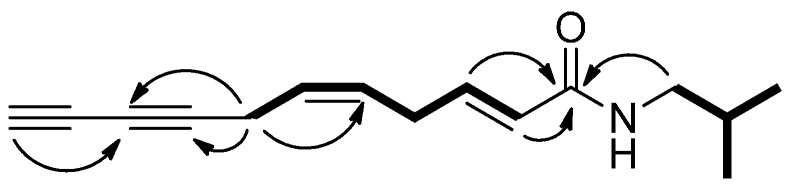
Selected COSY (bold line) and HMBC (arrow) correlations of **1**.

The plant materials of *Acmella oleracea* were collected in Taichung City, Taiwan. The flowers were extracted with ethanol. After removing ethanol under vacuum, the residue was partitioned between water and ethyl acetate to yield an ethyl acetate dissolvable extract. One new alkylamide, (2*E*,5*Z*)-*N*-isobutylundeca-2,5-diene-8,10-diynamide (**1**), and four known compounds (2*E*)-*N*-isobutyl-2-undecene-8,10-diynamide (**2**), (2*E*)-*N*-(2-methylbutyl)-2-undecene-8,10-diynamide (**3**), spilanthol (**4**), and (2*E*,6*Z*,8*E*)-*N*-(2-methylbutyl)-2,6,8-decatrienamide (**5**) were purified by repeated column chromatography form the extract (Scheme 1).

(2*E*,5*Z*)-*N*-Isobutylundeca-2,5-diene-8,10-diynamide (**1**) had a molecular formula of C_15_H_19_NO and seven degrees of unsaturation, as inferred from HRESIMS (*m*/*z* 252.1364 [M+Na]^+^) and ^13^C-NMR spectra. The UV absorption bands at 212, 264 and 279 nm implied that **1** could be an alkylamide [2]. The IR spectrum indicated the presence of secondary amide (3291, 1661, 1547 cm^−1^), and alkyne (2220 cm^−1^) functionalities. The ^1^H-NMR data of **1** clearly indicated the presences of two methyls (δ_H_ 0.92, 6H), two methines (δ_H_ 1.80 and 1.99), three aliphatic methylenes (δ_H_ 2.94, 3.01, and 3.15), and four olefinic methines (δ_H_ 5.57, 5.57, 5.79, and 6.82). The ^13^C-NMR and DEPT spectra of **1** demonstrated the presence of one amide carbonyl (δ_C_ 165.7), four olefinic methines (δ_C_ 124.4, 124.5, 128.0, and 141.0), one aliphatic methine (δ_C_ 28.6), four C-C triple bond carbons (δ_C_ 64.9, 65.2, 68.2, and 75.5), three aliphatic methylenes (δ_C_ 17.5, 29.4, and 46.9), and two methyls (δ_C_ 20.1). Comparing the ^1^H and ^13^C-NMR spectroscopic data of **1** with those of (2*E*)-*N*-isobutyl-2-undecene-8,10-diynamide (**2**), compound **1** possessed the same *N*-isobutyl and diynamide moieties except for an C-C double bond. The COSY spectrum (Figure 1) of **1** exhibited two ^1^H-^1^H coupling systems. One is H-2 (δ_H_ 5.79)/H-3 (δ_H_ 6.82)/H-4 (δ_H_ 2.94)/H-5 (δ_H_ 5.57)/H-6 (δ_H_ 5.57)/H-7 (δ_H_ 3.01), and the other is NH (δ_H_ 5.47)/H-1′(δ_H_ 3.15)/H-2′(δ_H_ 1.80)/H-3′ (δ_H_ 0.92) & H-4′(δ_H_ 0.92) (Figure 1). Both sequences were connected by the HMBC correlations from H-2, H-3 and H-1′ to C-1 (δ_C_ 165.7). A C-C double bond was assigned at C-5 and C-6 by virtue of the HMBC correlations from H-7 to C-5 (δ_C_ 124.5)/C-6 (δ_C_ 128.0). Moreover, the HMBC correlations from H-7 to C-8 (δ_C_ 75.5) and C-9 (δ_C_ 65.2) and H-11 (δ_H_ 1.99) to C-9 revealed a diyne moiety attached at C-7 (Table 1). The above 2D-NMR spectroscopic analysis identified **1** as an alkylamide with a new C-C double bond at C-5, and the structure of **1** was established as shown. 

A convenient method for concentrating the alkylamide-rich fraction was also developed by the following procedures. A. The ethanolic extract of the fresh flowers of *A. oleracea* (extract/plant material: *ca.* 6% *w*/*wet w*) was partitioned between EtOAc (EA) and H_2_O (1:1) to yield an EtOAc layer (*ca.* 22% *w*/*w* of ethanolic extract). B. The EtOAc layer was subjected to Silica diol gel (MB100-40/75) column chromatography eluting with *n*-hexane (H), H:EA 20:1, 10:1, 5:1, 1:1, and EtOAc. C. The alkylamide-rich fraction was isolated from the solvent system between H:EA 20:1 and 10:1. The alkylamide-rich fraction was yielded (*ca.* 32% *w*/*w* of EA layer).

**Table 1:** ^1^H (600 MHz) and ^13^C (150 MHz) NMR assignments and HMBC correlations of **1** (CDCl_3_).

NO.	δ_H_	δ_C_	HMBC
1		165.7 (s)	H-2, H-3, H-1′
2	5.79 (dt, 15.0, 1.8)	124.4 (d)	H-4
3	6.82 (dt, 15.0, 6.0)	141.0 (d)	H-4
4	2.94 (dt, 5.7, 1.8)	29.4 (t)	
5	5.57 (m)	128.0 (d)	H-4, H-7
6	5.57 (m)	124.5 (d)	H-7
7	3.01 (d, 4.2)	17.5 (t)	
8		75.5 (s)	H-7
9		65.2 (s)	H-7, H-11
10		68.2 (s)	
11	1.99 (t, 1.2)	64.9 (d)	
1′	3.15 (t, 6.0)	46.9 (t)	H-2′, H-3′
2′	1.80 (m)	28.6 (d)	H-3′
3′ and 4′	0.92 (d, 6.2)	20.1 (t)	
NH	5.47 (brs)		

**Figure 2:**
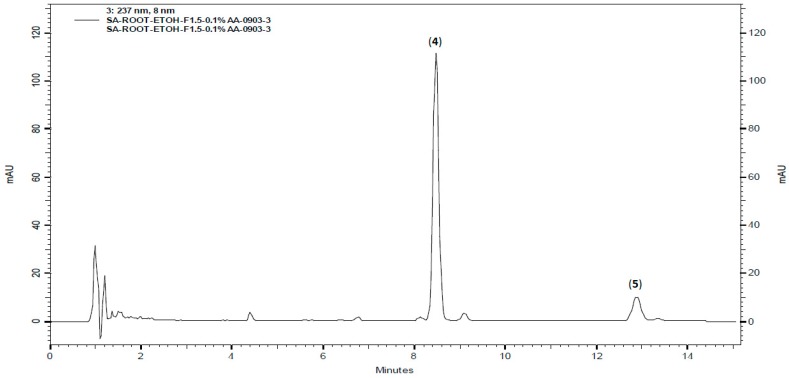
Qualitative HPLC profile of the crude extract of *A. oleracea* flowers shown the major compounds **4** and **5**.

To understand abundance and distribution of the bioactive alkylamides in this plant, HPLC-PDA was used to investigate on the subject. Separation on a reversed phase C-18 column (250 × 4.6 mm) with acetonitrile-H_2_O (45:55, 0.01−10.00 min, 50:50, 10.00−15.00 min, flow rate = 1.2 mL/min, 45 °C) as a solvent system provided good separation of the major alkylamides **4** and **5**. The fingerprint profile of the ethanolic extract of *A. oleracea* flowers carried out by the above condition is shown in Figure 2.

Calibration curves were established with five concentrations (12.5−200 μg/mL) of compounds **4** and **5** (see Experimental section). The linearity of the plot of concentration (x, μg/mL) for each compound versus peak area (y) was investigated. Under these analytical conditions, good linearities for all of the calibration curves were obtained (Table 2).

**Table 2:** Regression equations and retention times of compounds **4** and **5** determined for the HPLC assay.

Compound	Rt (min)	Regression equation	Linear Range (μg/mL)	R^2^
**4**	8.5	y = 41974x + 40516	12.5−200	0.9999
**5**	12.9	y = 2445.3x − 36077	12.5−200	0.9782

As indicated in Table 3, the predominated compound, spilanthol (**4**), was abundant and less-impure in the flowers (84.52 ± 0.81 mg/g of the ethanolic extract) than other parts of *A. oleracea*. The other important component, (2*E*,6*Z*,8*E*)-*N*-(2-methylbutyl)-2,6,8-decatrienamide (**5**), showed higher concentration in the aerial part.

**Table 3:** The average contents of compounds **4** and **5** from different parts of *A. oleracea*.

Compound (mg/g)	Plant part		
	Flower	Aerial part	Root
**4**	84.52 ± 0.81	56.60 ± 3.14	77.98 ± 0.13
**5**	5.82 ± 0.10	16.25 ± 1.89	12.91 ± 0.12

## 3. Experimental Section

### 3.1. General Experimental Procedures

Silica gel 60 (Merck) was used for column chromatography. The instrumentation for HPLC was composed of a Shimadzu LC-10AT pump and a Shimadzu SPD-20A UV-Vis detector (Shimadzu Inc., Kyoto, Japan). UV spectra were obtained using a Jasco UV-530 ultraviolet spectrophotometers. IR spectra were obtained on a Perkin Elmer system 2000 FT-IR spectrophotometer. Optical rotations were measured with a Jasco P-1020 digital polarimeter. NMR spectra were obtained by JEOL JNM ECS 400 MHz and Varian 600 MHz NMR. ESI-MS data were collected on a VG Biotech Quattro 5022 mass spectrometer. High-resolution ESI-MS data were obtained on a Bruker APEX II spectrometer (FT-ICR/MS, FTMS) (Bruker Daltonics Inc., Billerica, MA, USA).

### 3.2. Plant Material

The specimens of *Acmella oleracea* were collected in Taichung City, Taiwan, in June, 2011. The plant material was identified by one of the authors, Rosa Huang Liu. A voucher specimen (code no. KMU-Acmella 1) was deposited in the Graduate Institute of Natural Products, College of Pharmacy, Kaohsiung Medical University, Kaohsiung, Taiwan.

### 3.3. Extraction and Isolation

The fresh flowers of *A. oleracea* (4.5 kg) were extracted with 95% aqueous EtOH at room temperature and then concentrated under reduced pressure. The crude extract (285.0 g) was partitioned between EtOAc and H_2_O (1:1) to yield an EtOAc layer (63.3 g). The EtOAc layer was subjected to Silica diol gel (MB100-40/75) column chromatography under a gradient elution of (*n*-hexane/EtOAc/MeOH) to yield 26 fractions (F-1 to F-26). F-11 (20.8 g) was eluted with EtOAc-MeOH (1:1) by a LH-20 column to obtain four sub-fractions (F11-1 to F11-4). Fraction F11-4 (125.0 mg) was further purified by a RP-HPLC (C_18_) (85% MeOH, isocratic, flow rate 2.0 mL/min, UV 237 nm, 5 µm, Thermo Hypersil, 250 × 10.0 mm column) to obtain **4** (13.2 mg) and **5** (17.1 mg). Fraction F13 (207.6 mg) was purified by a RP-HPLC (C_18_) (70% MeOH, isocratic, flow rate 2.0 mL/min, UV 237 nm, 5 µm, Thermo Hypersil, 250 × 10.0 mm column) to yield **1** (2.5 mg), **2** (31.5 mg), and **3** (2.1 mg).

### 3.4. Spectral Data

(2*E*,5*Z*)-*N*-Isobutylundeca-2,5-diene-8,10-diynamide (**1**): Pale yellow oil; UV (MeOH): 221 (3.88); IR (neat): 2956, 2354, 2325, 1661, 1641, and 1547 cm^−^^1^; ^1^H-NMR (CDCl_3_, 600 MHz) and ^13^C-NMR (CDCl_3_, 150 MHz) see Table 1; HR-ESI-MS *m*/*z* 252.1364 ([M + Na]^+^, calcd. for C_15_H_19_NONa 252.1364).

### 3.5. Crude Samples Prepared from Different Parts of A. oleracea for Qualitative and Quantitative Analysis

Flesh flowers, aerial parts, and roots were ground and extracted with ethanol at 24–25 °C. All extracted solutions were evaporated under reduced pressure to give three extracts. Each dry extract (1.0 mg) was dissolved in MeOH (1.0 mL), filtered on a pre-column and injected to HPLC (each injection was 10 μL). 

### 3.6. Analytical HPLC

HPLC analyses were executed on a Shimadzu model LC-10AT HPLC (Japan) equipped with SPD-M10A diode array detector. The wave length of detector was set at 237 nm. Data acquisition and quantification were performed by the Shimadzu Class-VP software (version: 6.12SP5). Chromatography was carried out on an Agilent Poroshell 120 (250 × 4.6 mm) column. The solvents were filtered through a 0.45 μm filter prior and the total HPLC running time for the assay was 15 minutes.

### 3.7. Calibration

In the standard HPLC chromatogram, five different concentrations of compounds **4** and **5** in the linear range (12.5−200 μg/mL) were prepared in MeOH, respectively. Three replicates (n = 3) of each concentration were subjected to HPLC. The methods of the experimental section 3.5−3.7 were performed according to our previous study [11].

## 4. Conclusions

According to literatures, these types of alkylamides were found among the plants belonging to the families of Asteraceae. They showed a series of bioactivities and now are very important in pharmaceutical and cosmetic industry. For example, alkylamides from *Echinacea* are merchantable supplementary food. In our phytochemical investigation, a new alkylamide named (2*E*,5*Z*)-*N*-isobutylundeca-2,5-diene-8,10-diynamide (**1**) was successfully purified and identified. Besides the new alkylamide discovery, the method for efficient extraction, concentration and rapid analysis of alkylamides from different plant parts of *Acmella oleracea* was established. These analytical studies provide necessary information for quality control assessment of the target plant.

## Supplementary Materials

Supplementary materials can be accessed at: http://www.mdpi.com/1420-3049/20/04/6970/s1.
