# Supplementary material for: Alkylamides of Acmella oleracea
Source: Molecules. 2015 Apr 16;20(4):6970–7. doi: 10.3390/molecules20046970 (PMC6272129; doi:10.3390/molecules20046970)
Supplement: Supplementary file 1 [file molecules-20-06970-s001.pdf]

Supplementary Information

Contents

Figure S1. ^{1}H-NMR Spectrum of **1** in CD_3OD S2
Figure S2. ^{13}C-NMR Spectrum of **1** in CD_3OD S3
Figure S3. DEPT Spectrum of **1** in CD_3OD S4
Figure S4. COSY Spectrum of **1** in CD_3OD S5
Figure S5. HMQC Spectrum of **1** in CD_3OD S6
Figure S6. HMBC Spectrum of **1** in CD_3OD S7
Figure S7. NOESY Spectrum of **1** in CD_3OD S8

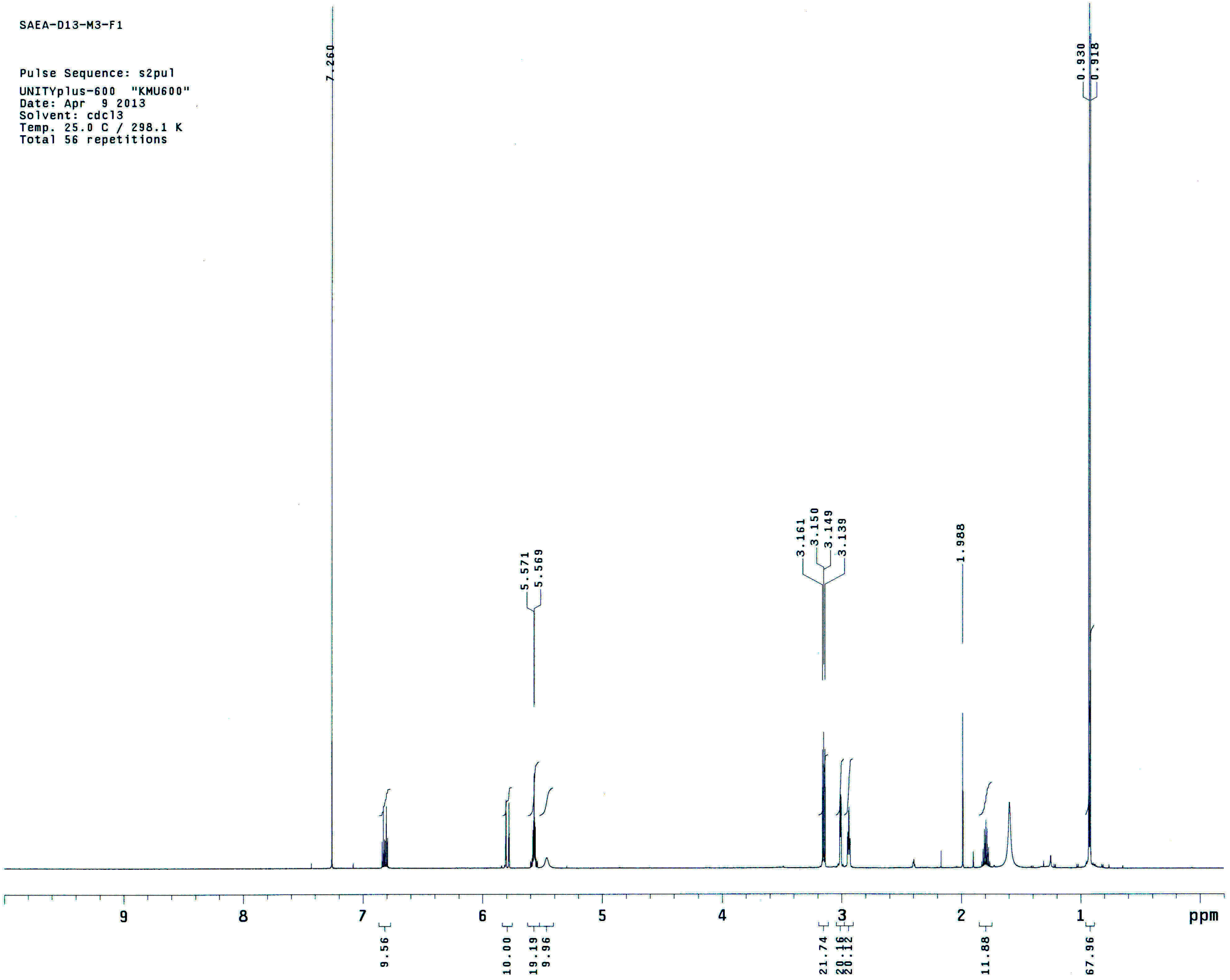

Figure S1. ^{1}H-NMR Spectrum of **1** in $CDCl_3$.

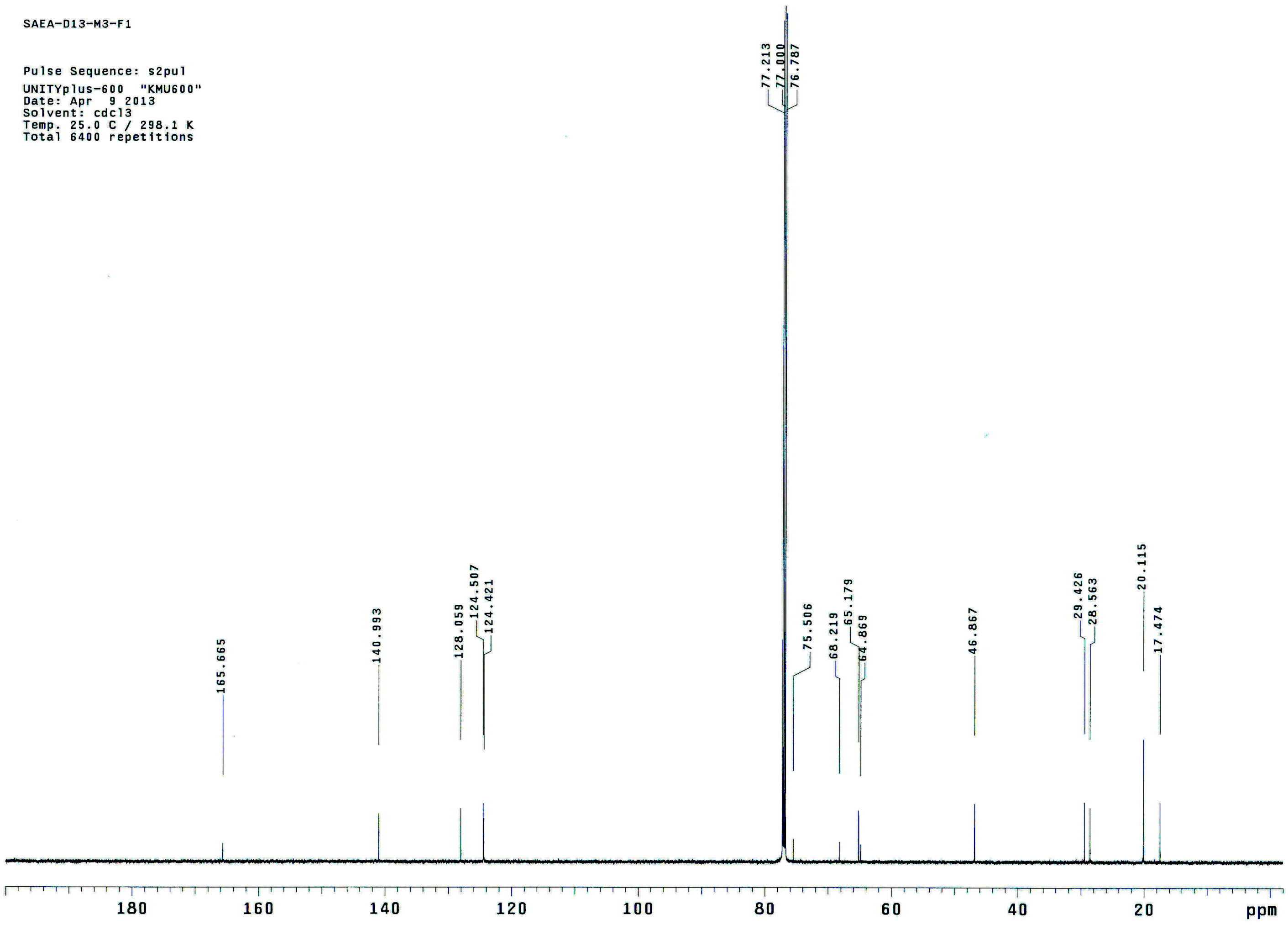

Figure S2. ^{13}C-NMR Spectrum of **1** in $CDCl_3$.

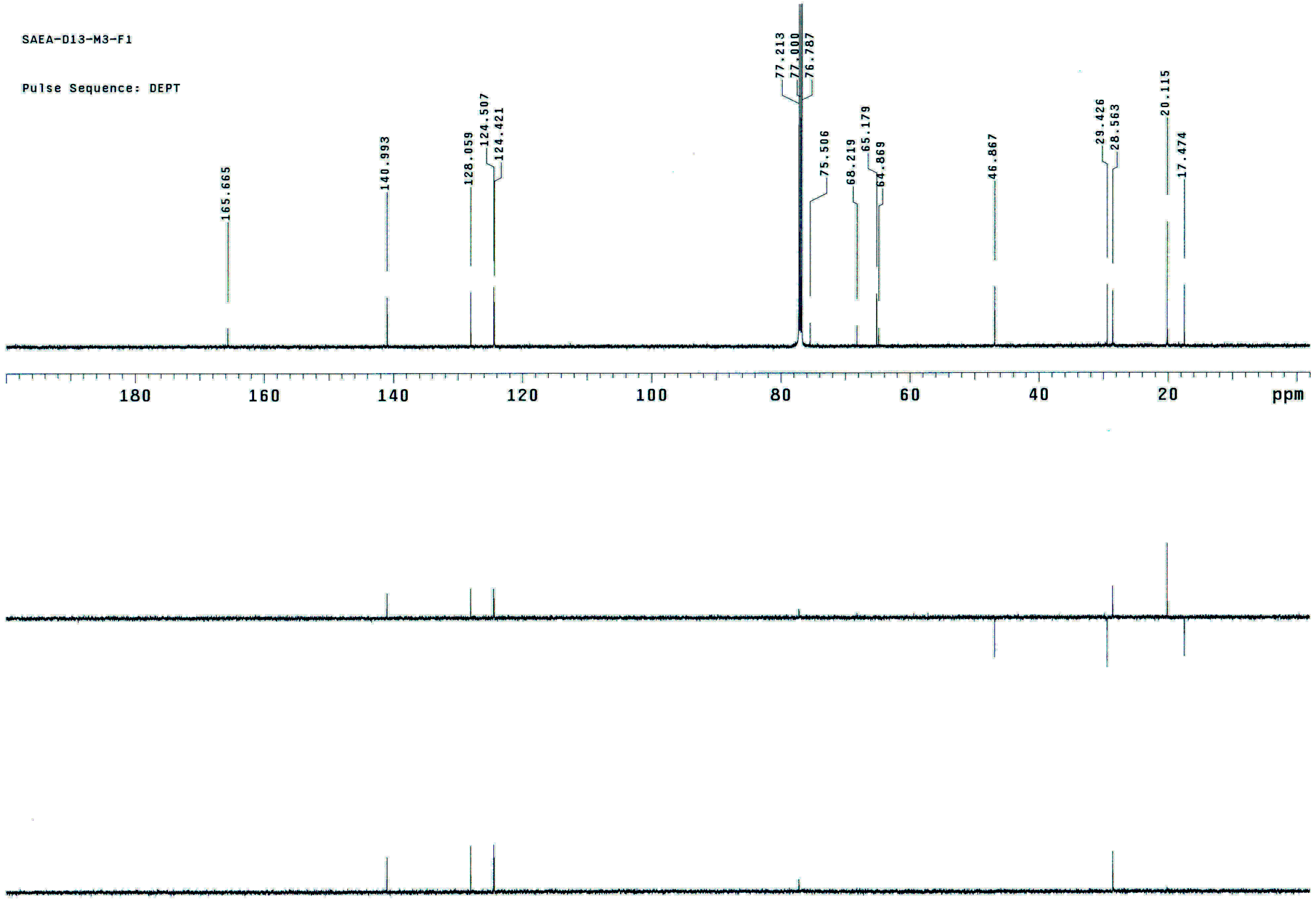

Figure S3. DEPT Spectrum of **1** in $CDCl_3$.

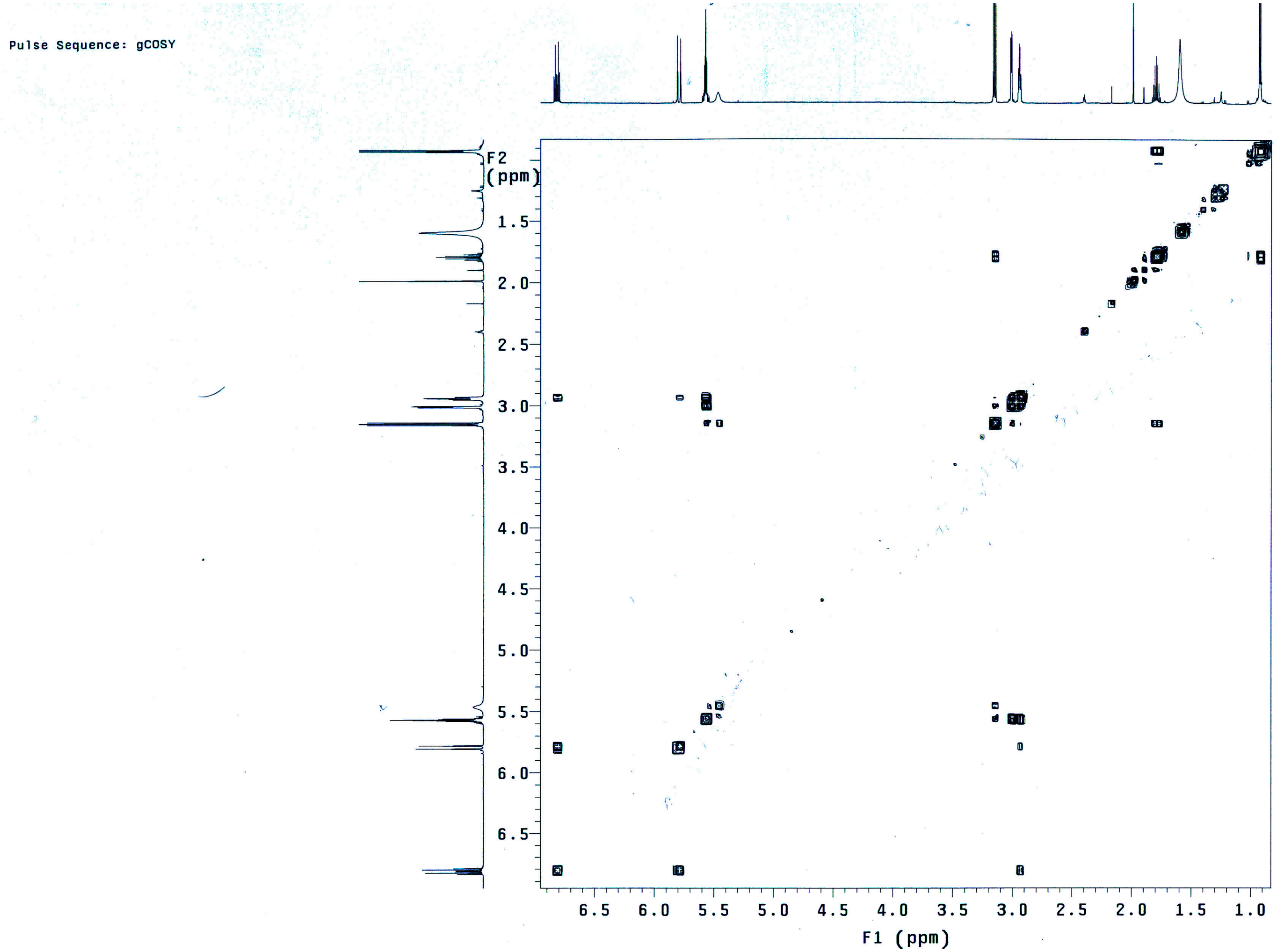

Figure S4. COSY Spectrum of **1** in $CDCl_3$.

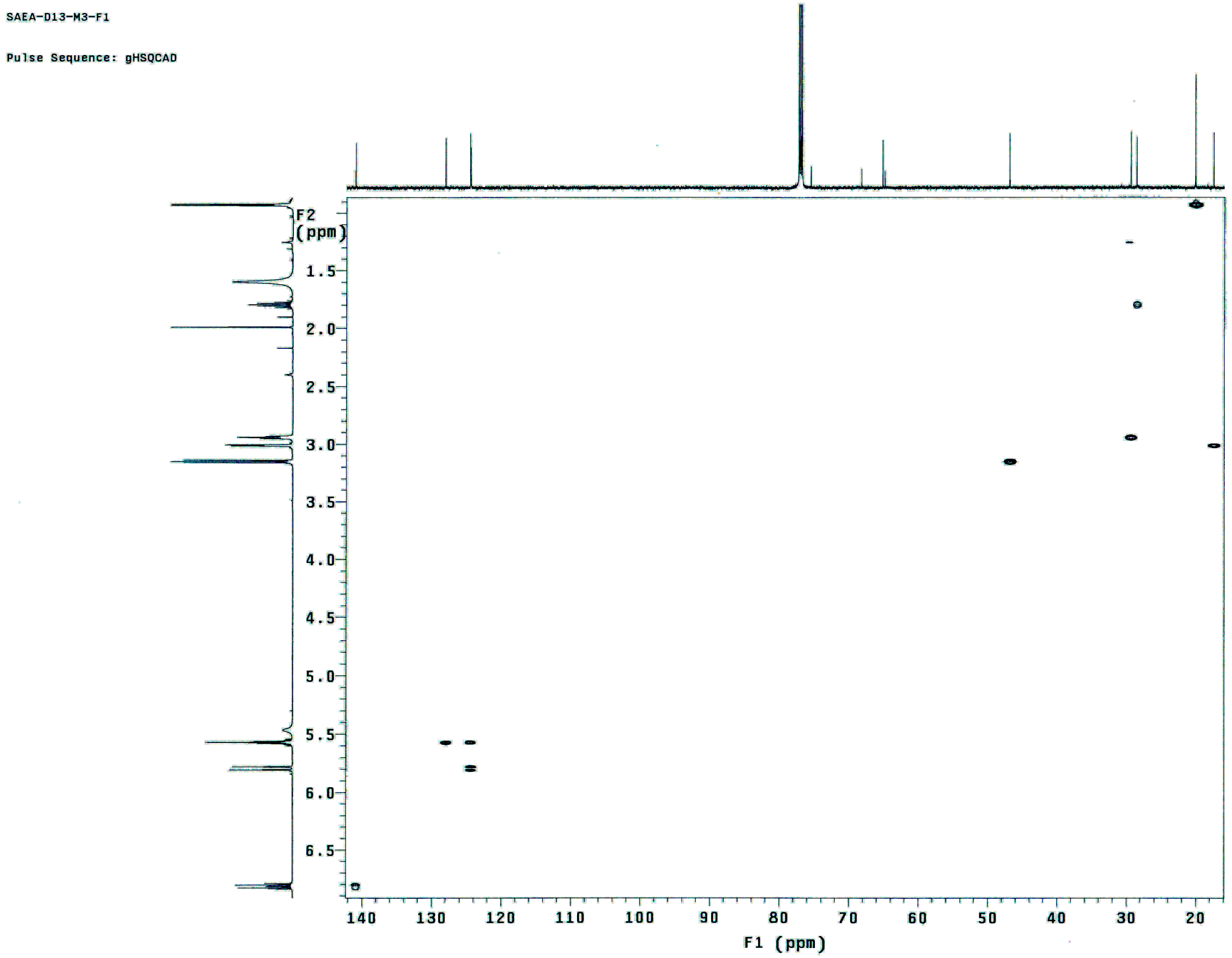

Figure S5. HMQC Spectrum of **1** in $CDCl_3$.

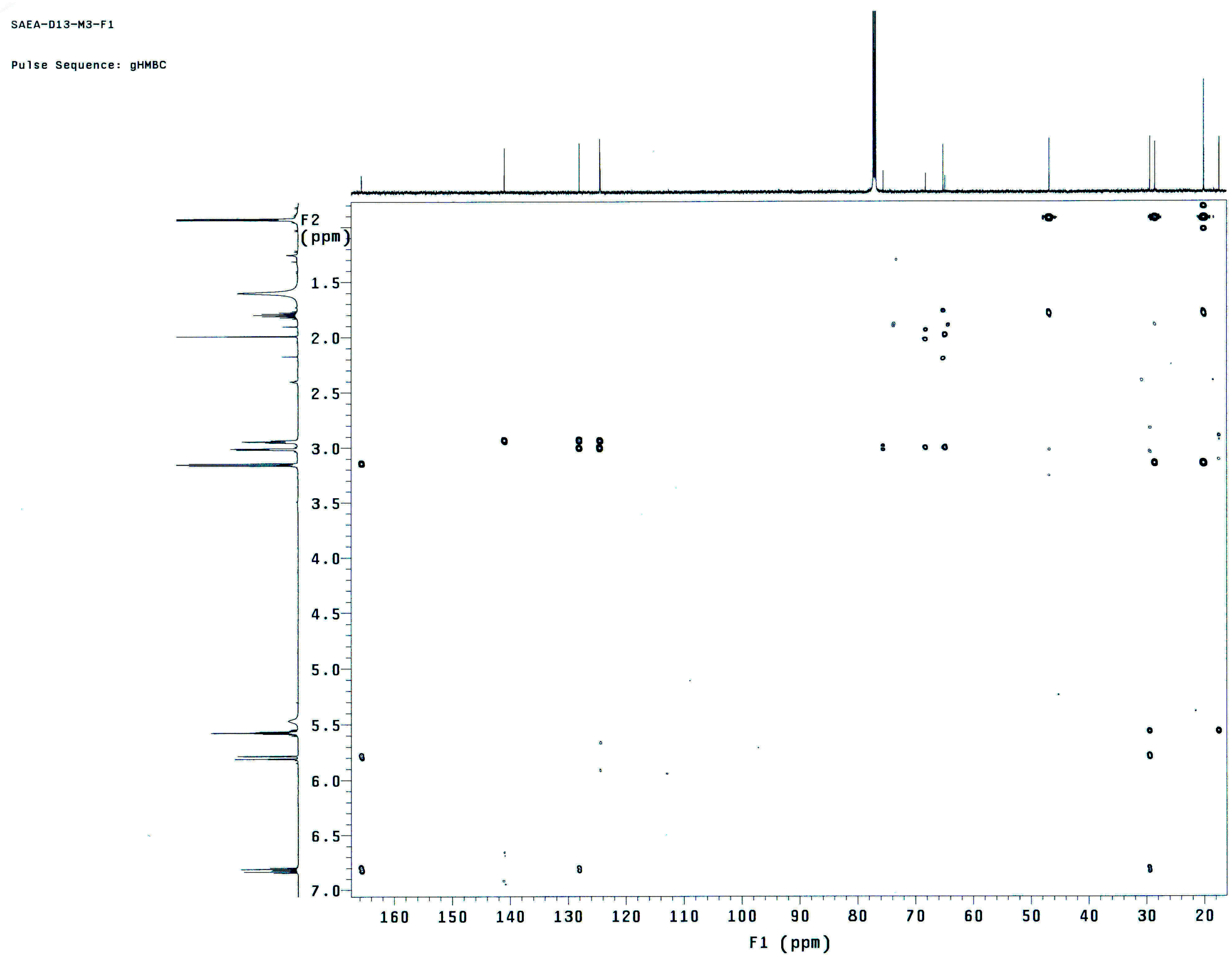

Figure S6. HMBC Spectrum of **1** in $CDCl_3$.

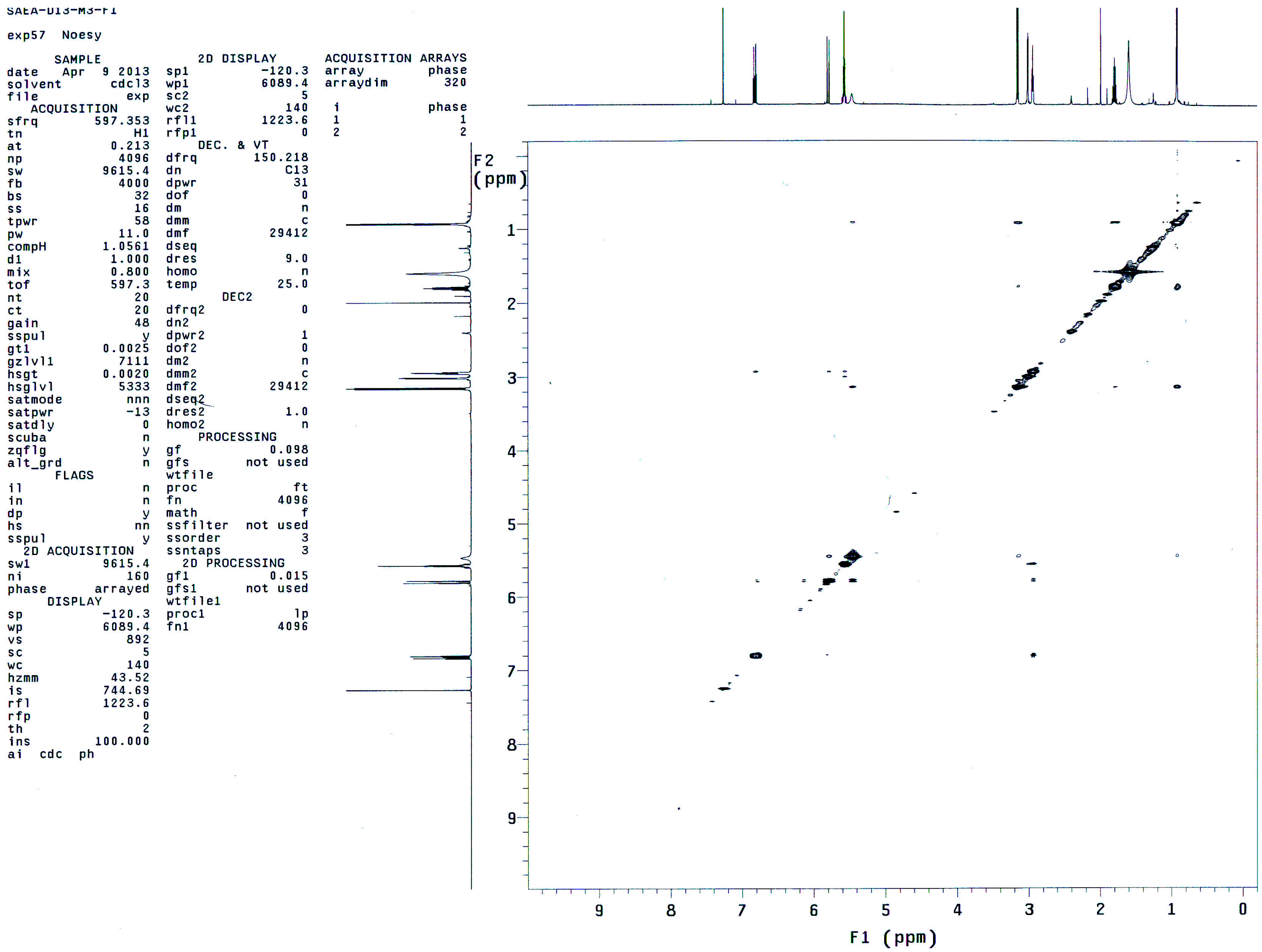

Figure S7. NOESY Spectrum of **1** in $CDCl_3$.
